# Real-time prediction of atrial fibrillation in intensive care unit: a meta-learning approach

**DOI:** 10.1093/jamiaopen/ooag110

**Published:** 2026-07-11

**Authors:** Mehran Moazeni, Sebastian Mildiner Moraga, Maaike Wösten, Paul Elbers, Patrick Thoral, Linda Wilhelmina Van Laake, Folkert Wouter Asselbergs, Emmeke Aarts

**Affiliations:** Department of Methodology and Statistics, Utrecht University, Padualaan 14, 3584 CH Utrecht, The Netherlands; Department of Methodology and Statistics, Utrecht University, Padualaan 14, 3584 CH Utrecht, The Netherlands; Department of Cardiology, University Medical Centre Utrecht, Utrecht University, Heidelberglaan 100, 3584 CX Utrecht, The Netherlands; Computational Imaging Group for MRI Therapy & Diagnostics, University Medical Centre Utrecht, Heidelberglaan 100, 3584 CX Utrecht, The Netherlands; Department of Intensive Care Medicine, Center for Critical Care Computational Intelligence, Amsterdam Medical Data Science, Amsterdam Public Health, Amsterdam Institute for Immunity and Infectious Diseases, Amsterdam Cardiovascular Science, Amsterdam UMC, Vrije Universiteit, University of Amsterdam, De Boelelaan 1117, 1081 HV Amsterdam, The Netherlands; Department of Intensive Care Medicine, Center for Critical Care Computational Intelligence, Amsterdam Medical Data Science, Amsterdam Public Health, Amsterdam Institute for Immunity and Infectious Diseases, Amsterdam Cardiovascular Science, Amsterdam UMC, Vrije Universiteit, University of Amsterdam, De Boelelaan 1117, 1081 HV Amsterdam, The Netherlands; Department of Cardiology, University Medical Centre Utrecht, Utrecht University, Heidelberglaan 100, 3584 CX Utrecht, The Netherlands; The National Institute for Health and Care Research (NIHR), University College London Biomedical Research Centre (BRC), University College London, Gower Street, London, WC1E 6BT, United Kingdom; Health Data Research United Kingdom and Institute of Health Informatics, University College London, 222 Euston Road, London, NW1 2DA, United Kingdom; Department of Cardiology, Amsterdam Cardiovascular Sciences, Amsterdam University Medical Centre, University of Amsterdam, Meibergdreef 9, 1105 AZ Amsterdam, The Netherlands; Department of Methodology and Statistics, Utrecht University, Padualaan 14, 3584 CH Utrecht, The Netherlands

**Keywords:** atrial fibrillation (AF), intensive-care unit (ICU), early prediction, long short-term memory (LSTM), SHAP values

## Abstract

**Objective:**

Atrial fibrillation (AF) is a common arrhythmia affecting a large fraction of patients in intensive care units (ICUs). Predicting patients at risk of AF in the ICU is a challenging task and is not commonly practiced but could have clinical implications considering that AF is a proxy for poorer outcomes. Our goal is to develop a real-time AF prediction model using ICU numerical data to improve alarm quality, helping clinicians to identify at-risk patients and intervene before AF onset, thereby potentially improving clinical outcomes.

**Methods:**

We employed AmsterdamUMCdb, Europe’s first openly accessible ICU dataset. The dataset includes static features, including demographics, as well as dynamic bedside monitoring data, like blood pressure and respiratory rate, along with detailed records of medication and fluid administration. To enhance generalizability across diverse ICU patients, we trained a long short-term memory (LSTM) model combined with a Model-Agnostic Meta-Learning (MAML) approach. Model performance was tested on an imbalanced test set, reflective of the real-world ratio of AF to non-AF patients. Direct external validation was performed using the MIMIC-IV database to test generalizability across different clinical settings.

**Results:**

The LSTM-MAML model achieved an AUC of 0.92, accuracy of 0.89, and precision of 0.29 on the internal validation set. In external validation with MIMIC-IV, it showed near-equivalent performance with an AUC of 0.89, accuracy of 0.85, and precision of 0.18.

**Conclusions:**

The real-time prediction model demonstrated predictive value for AF and has potential for future clinical benefits. However, for clinical implementation, further refinement is necessary to improve its performance in identifying patients at risk for AF.

## Introduction

New-onset atrial fibrillation (AF) is a common complication in critically ill patients and affects up to 44% of those admitted to the ICU.^1–3^ While some AF risk factors cannot be modified (e.g., age), early prediction of AF in the ICU enables clinicians to intervene promptly (e.g.,, adjusting medications or managing conditions like infection). Timely interventions may help reduce negative hospital outcomes such as short- and long-term increases in the risk of stroke, heart failure, and death.^1^^,^^4^

Foreseeing AF ahead of time is challenging and requires continuous monitoring by ICU staff, which has led researchers to explore the use of electrocardiogram (ECG) signals and machine learning models for AF prediction.^4^^,^^5^ While ECG remains the gold standard for AF detection in the clinic and ECG-based algorithms have demonstrated strong accuracy in previous studies, using high-frequency ECG waveforms for prediction at scale often requires expert interpretation and annotation, particularly when labels must be derived from several hours of ECG data prior to the event (e.g., 24 hours) to enable true prospective prediction rather than retrospective detection. The need for specialized expertise limits the size and diversity of patient groups that can be studied because the large volume of ECG data requires significant time and effort to analyze manually. Additionally, ECG signals are sensitive to patient movement, which can lead to a high number of false positive alarms in predictions. As a result, other studies have focused on utilizing numerical ICU data (e.g., medications, bed-side data, and fluids administration) to predict AF.^5–7^

AF prediction using numerical data can be divided into 2 approaches. One approach is real-time prediction, generating risk scores for each measurement, while the other is patient-wise prediction, assigning a single risk score for the ICU stay. Recall and precision in prior studies range from 15% to 75% and 7% to 74%, respectively, with cases of higher recall often having lower precision, and vice versa.^5^^,^^6^^,^^7^^,^^8^^,^^9^ Additionally, some research has created semi-real-time models with time periods (such as 6-24 hours), but additional development is required to improve the balance between true positive and false positive alarms in real-time risk scores.^5^

Our objective is to develop a real-time prediction model that predicts each hour as AF or No-AF during a patient’s ICU stay. Using numerical data such as physiological signals, medication, or fluid administration, we aim to improve model performance metrics such as recall and precision. By incorporating meta-learning, the model can adapt to each patient’s unique data, enhancing generalization across diverse cases. This approach also allows the model to make accurate predictions without the need for additional fine-tuning, saving time and resources. We use long short-term memory (LSTM) networks to recognize temporal patterns in patient time-series data, while meta-learning is designed to address inter-patient variability, improving the model’s potential to generalize across diverse patients.

## Methods

Our model development and internal validation utilized the Amsterdam University Medical Centers ICU database (AmsterdamUMCdb—v1.0.02). The database contains information from a 32-bed combined surgical-medical academic intensive care unit (ICU) and a 12-bed intermediate care facility.^9^ For patients who experienced AF during their ICU stay, the primary endpoint was the first time AF was recorded by nursing staff after at least 1 sinus rhythm registration. AF onset during the ICU stay was identified from charted diagnoses based on ECG monitoring, while continuous ECG waveforms were not available; ECG thus defines the outcome, but our prediction model uses only routinely collected numerical data as predictors. The data processing and analysis pipeline is visualized in Figure 2. The figure depicts the stages from raw data acquisition to the final model evaluation. In the paragraphs below, each of these stages is briefly described.

### Preprocessing

We selected patients with a minimum length of stay (LOS) of 6 hours and a maximum of 300 hours. Thresholds were chosen to provide adequate data for training and testing while also minimizing variability between patients based on LOS.

To ensure consistency in the frequency of consecutive measurements, we standardized the frequency by averaging the measurements within each hour to achieve 1 measurement per hour (ie, interval harmonization). Additionally, we introduced 2 new features into the model: one to represent the number of measurements averaged and another to indicate their standard deviation.

Missing values for the dosage of administered medications and fluids were set to 0, indicating that no prescription was made at that specific time point. For handling missing data from device-generated measurements and manually entered observations, 2 approaches were applied. If the missing rate for a variable was below 25%, we used Multiple Imputation by Chained Equations (MICE), which relies solely on relationships present in the observed data. When missingness exceeded 25%, we applied forward filling on a per-patient basis, carrying the last observed value forward until a new measurement became available (details in Section SA.2). This approach preserves patient-specific temporal structure and avoids introducing values derived from other patients. If all values for a patient were missing for a given feature, the population-level expected value was used as a neutral baseline, following the approach of Hyland et al.^10^ Because many ICU measurements are recorded only when clinically indicated, retaining variables with partial missingness preserves informative clinical context that would otherwise be lost by exclusion. For values for which minimum and maximum physiologically expected ranges were provided, we removed artifacts falling outside these limits (see Table S1). For external validation, only features present in both AmsterdamUMCdb and MIMIC-IV were included in model training and testing (details in Table S6).

### Pairing AF and non-AF admissions based on ICU stay

Patients who develop AF typically have longer ICU stays, introducing an additional layer of imbalance to the dataset. To address this issue and facilitate a more direct comparison between AF and non-AF patients, we constructed a paired cohort of AF and non-AF admissions following the case–control temporal alignment procedure described by Verhaeghe et al.^5^ Importantly, this procedure does not constitute propensity-score matching. Instead, its purpose is to ensure that each AF admission is compared with a non-AF admission for which the AF diagnosis time is a feasible prediction point (ie, before ICU discharge of the non-AF patient). Each AF patient was paired one-to-one with a non-AF patient, based on the relative time from ICU admission, ensuring that the AF diagnosis occurred within a time window shorter than the time until ICU discharge of the matched non-AF patient. Both admissions shared a single prediction point, termed the surrogate AF prediction point. For the non-AF patient, the surrogate AF prediction point was used as the primary endpoint. This matching process was completed prior to splitting the data into training and testing sets.

### Labeling

The last 2 hours before the events (e.g., AF onset for AF patients and surrogate AF prediction point for non-AF patients) were excluded from the analysis (see Figure 2). This period, termed the buffer window, excludes the final 2 hours before the event from model training and evaluation, thus providing a potential window for clinical interventions aimed at preventing AF. Within the analyzed time frame, the final 24 hours was identified as the “Pre-AF window.” This window is crucial for detecting AF, highlighting the period where the model focuses on identifying the onset of AF. Labels were defined as binary values, where 1 indicated being at risk for AF and 0 indicated no AF risk. In the Pre-AF window, for AF patients, depending on their length of stay (LOS), measurements may be either all 1s or a mix of some 0s and 1s. For non-AF patients, all measurements were 0, as these patients do not develop AF. All measurements prior to the Pre-AF window were assigned to the “No-AF window,” where labels were set to 0 for both patient groups.

### Model development

Our study employs the Model-Agnostic Meta-Learning (MAML) approach.^11^ MAML aims to broaden the model’s adaptability to various tasks, thereby enhancing its effectiveness in identifying heterogeneous patterns in data. In our study, a “task” is defined as a combination of sampled AF and non-AF patients from the original dataset, presenting the model with a learning challenge to distinguish between time-series data of the 2 patient groups.

Each task consists of a support set and a query set:

- **Support set**  (S): The support set includes labeled data for initial model fitting to the task, comprising full sequence data from $\left(N_{1}\right)$ patients with AF and $\left(N_{2}\right)$ non-AF patients. This set trains the model to identify distinguishing features and patterns between different patients.
- **Query set**  (Q): The query set assesses the model’s fitting, containing data from $\left(M_{1}\right)$ AF patients and $\left(M_{2}\right)$ non-AF patients, all previously unseen in the support set. This set tests the model’s learning and generalization capability.

At the heart of our methodology lies the LSTM neural network, serving as the learner. The learner is directly trained on a given task’s data to make predictions (aka *inner loop updates*). To achieve optimal results, we applied several different models, with the LSTM model providing the best performance (see Table S8). Notably, our study incorporates an imbalance-aware optimization strategy through the Combo Loss, a combination of Binary Cross-Entropy (BCE) and Dice lossto better account for the imbalanced class distribution: the Dice component emphasizes overlap between predicted and true AF events, while the BCE component stabilizes optimization, together encouraging the model to maintain sensitivity to AF events despite their lower prevalence.

Once the inner loop updates are finished, the MAML framework enables the model to adjust parameters to a new set of patient data, instead of specializing in a single, specific training dataset. This is achieved by monitoring the loss function values on the query set, referred to as *outer loop updates*. The primary goal of this step is to generalize the base learner parameters across different populations. The process of selecting the optimized hyperparameters for this study is thoroughly explained in Section SA.8. To further ensure robust and reliable results, we employed 5-fold cross-validation.

### Feature selection and evaluation

We selected 150 (available in Supplemental Table 2-5) features from the AmsterdamUMCdb dataset out of the initial 282 potential features identified by Verhaeghe et al.^5^ This selection ensures a comparable feature set to that used in prior studies, enabling consistency in analysis and facilitating meaningful comparisons between our findings and existing literature. Features with fewer than 250 occurrences or more than 99% missing data were excluded, following the criteria outlined by Verhaeghe et al.^5^ Additionally, we included auxiliary features as stated in Section 4.1. For external validation, the model was first trained on the AmsterdamUMCdb dataset and then directly applied to the MIMIC-IV dataset for comparison. Only the matched features between the 2 datasets were used for this process.

The models were constructed using Python, version 3.9.16. For the MAML model, the learn2learn package was used alongside PyTorch’s LSTM module (PyTorch version 2.1).^12^ The output from the models was transformed using a sigmoid function to yield probability scores that range from 0 to 1. A threshold of 0.5 was then applied to the AF risk scores to trigger alerts. The first alert is treated as a precautionary warning, so it is not categorized as either a true positive or a false positive. Should another alert occur within the next hour, it is recognized as an alarm, and any further alarms are treated similarly. This precautionary mechanism is employed once in the Pre-AF window and again in the Non-AF window, aiming to enhance understanding in both periods.

The performance of the model was assessed using various metrics, including precision, recall, F1 score, Area Under the Curve (AUC), G-mean (the geometric mean of recalls for AF and non-AF patients), and accuracy, drawn from a 20% held-out test set of patients. The model was tested in 2 settings: a balanced test set with an equal number of AF and non-AF patients, and an unbalanced test set that reflected real-world conditions, with the ratio of AF to non-AF patients set to 0.07.

### SHAP analysis

To illuminate the mechanisms and outcomes of the final models, SHAP analysis was performed. Each SHAP value is linked to a specific feature within a data sample, clearly indicating the impact of that feature on the model’s prediction in comparison to the model’s average prediction across all data samples.

## Results

**Figure 1.**
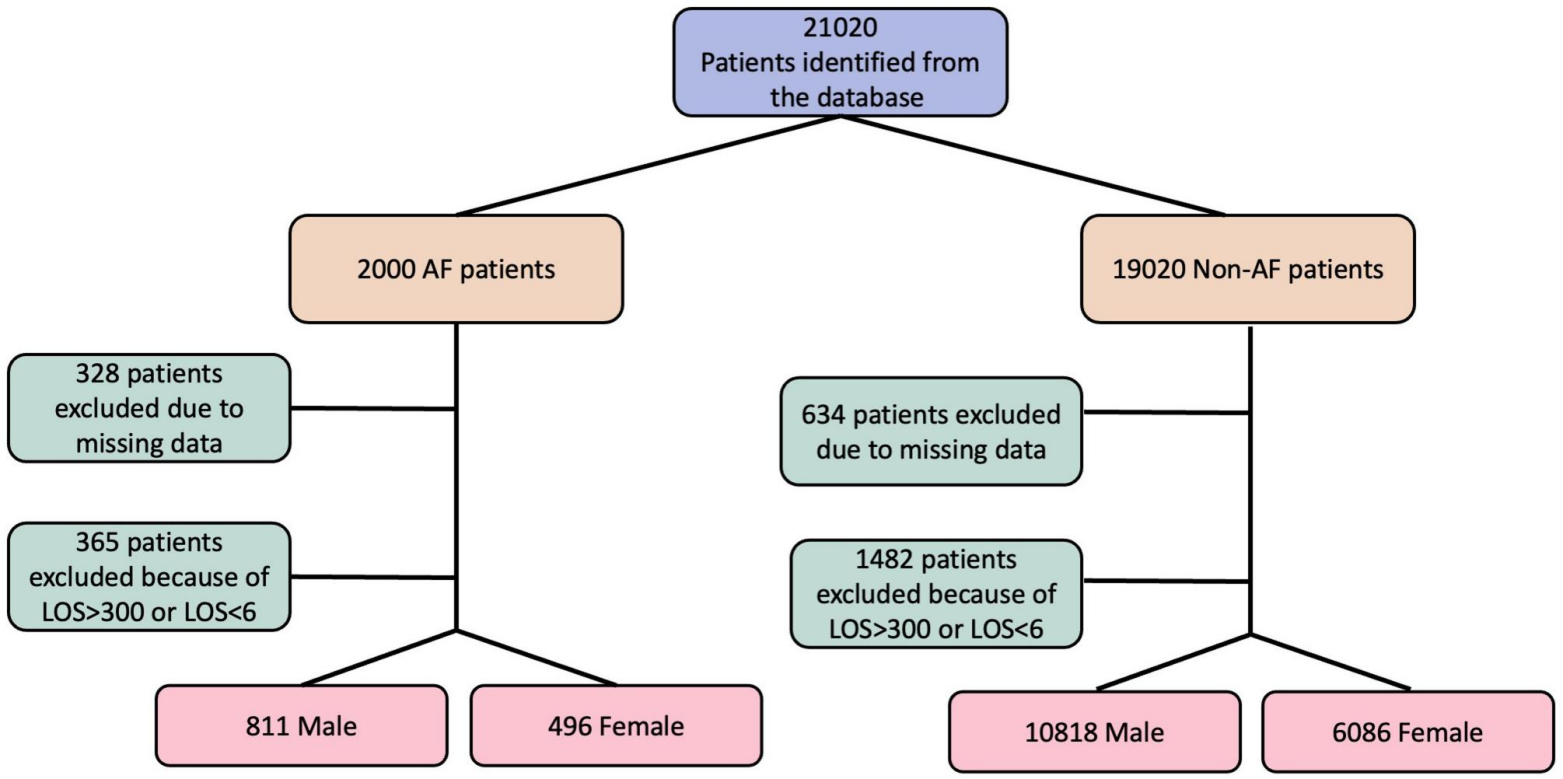
Flow diagram illustrating inclusion and exclusion criteria and final number of patients included in the study.

From the AmsterdamUMCdb dataset, out of 4086 patients diagnosed with AF and 19 020 without AF, we included 1307 AF patients and 16 904 non-AF patients in our study. Patients were excluded based on the LOS threshold explained in Section 2 (with further details shown in Figure 1). The baseline characteristics of the included patients are presented in Table 1. Twenty percent of these patients were allocated to the test set, consisting of 261 AF patients and 3389 non-AF patients, while the remaining patients participated in the model training.

**Figure 2.**
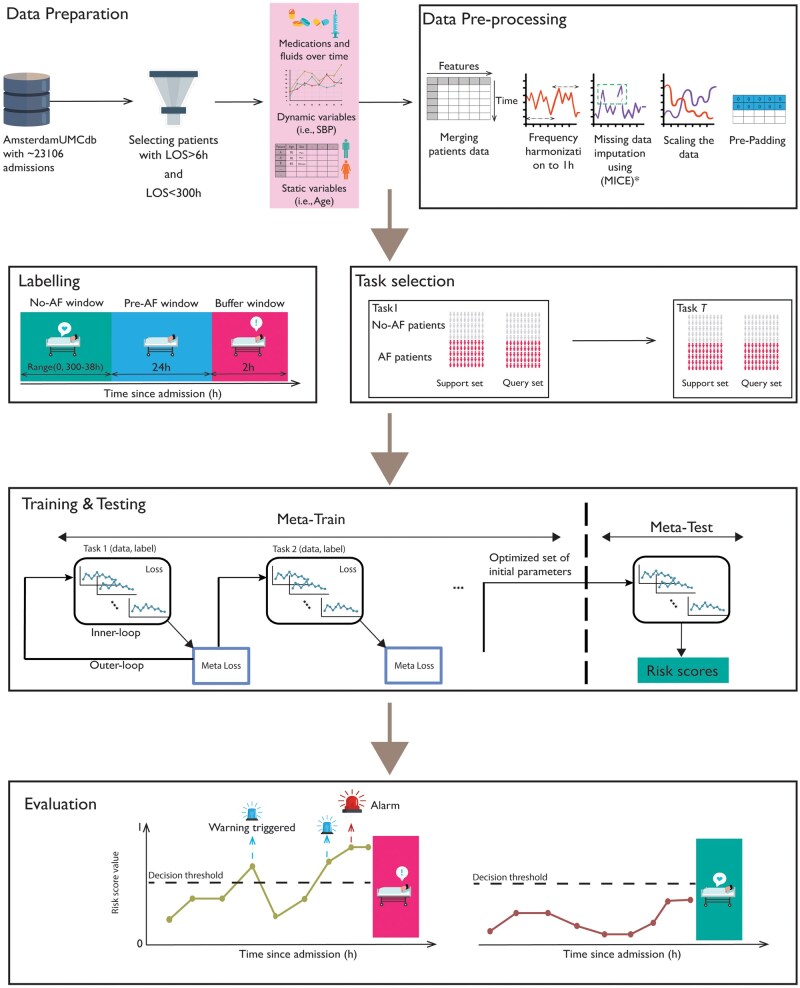
Overview of the meta-learning pipeline for AF prediction. In the data preparation step, patients data filtered by a minimum and a maximum LOS value. Various variables, including dosage of medications and fluids administered during treatment, were retrieved. Additionally, static variables were retrieved along with other dynamic features such as heart rate, blood pressure, and more. In the **Data pre-processing**, dynamic and static features are merged together, and then frequency of measurements harmonized so every hour each patient has up to 1 measurement per hour. Next, missing data imputed and data scaled and padded for next steps. **Labeling** involves assigning an AF label to the 24-hour period leading up to the onset of AF, while excluding data from the last 2 hours as a buffer window. This strategy enables the model to predict the onset of AF at least 2 hours in advance. In the **Task selection** step, patients were sampled from both AF and non-AF patients and grouped in tasks for training. The **Training and Testing** phase is structured around a meta-learning approach. It consists of an *inner-loop* and *outer-loop* learning process. In the *inner-loop*, the model is trained on the data (Task 1, Task 2, etc.) with an immediate loss calculation, whereas in the *outer-loop*, meta parameters are optimized based on the meta loss aggregated from all tasks. In the **Evaluation** step, the model’s predictions are analyzed over time from admission onward, to determine their risk level. If the risk scores surpass a predefined decision threshold, a warning or alert is triggered, indicating a high risk of AF. The warning system triggers alarm if 2 consecutive measurements exceed the decision threshold, otherwise silences the alert as a warning. MICE = Multiple Imputation by Chained Equations.

**Table 1.** Baseline characteristics of patients with and without AF for AmsterdamUMCdb and MIMIC datasets.

	**AmsterdamUMCdb**		**MIMIC**	
	AF patients	Non-AF patients	AF patients	Non-AF patients
**Cohort size**	1307	16 904	628	8971
**Age (years)**	70.2 [69.0-75.0]	61.0 [55.0-75.0]	69.1 [65.0-75.0]	63.2 [50.0-73.0]
**Gender (male %)**	807 (61.8%)	10 940 (64.2%)	379 (60.4%)	5086 (56.7%)
**BMI**	26.5 [23.5-27.4]	25.4 [23.4-27.1]	29.3 [22.4-34.7]	28.9 [22.6-32.2]
**Weight (kg)**	77.4 [65.0-85.0]	77.7 [65.0-85.0]	81.7 [65.0 85.0]	77.1 [65.0-85.0]
**Sepsis**	298 (22.8%)	1787 (10.5%)	123 (19.6%)	2296 (25.6%)
**Cardiac surgery**	335 (25.6%)	5039 (30.0%)	241 (38.4%)	2852 (31.8%)
**SOFA (first 24 hour)**	8.4 [6.0-11.0]	5.2 [3.0-8.0]	5.5[3.0-7.0]	3.6 [1.0-5.0]
**APACHE II (first 24 hour)**	22 [17.0-26.0]	16.6 [12.0-20.0]	/	/
**ICU LOS (days)**	14.3 [3.8-18.9]	3.0 [1.3-2.4]	8.1 [3.1-10.2]	3.8 [1.5-3.9]
**ICU survival rate**	46.4%	73.4%	79.1%	88.4%

Numbers in brackets indicate IQR.

In the external validation, there were initially 44 735 non-AF patients and 681 AF patients. To maintain the same AF to non-AF ratio, we down-sampled the non-AF patients. This resulted in a test set of 681 AF patients and 8971 non-AF patients (see Table 1). AF patients on average had an LOS of 86.4 hours while non-AF patient had an LOS of 76.5 hours.

### Model outcome

Tables 2 and 3 showcase the effectiveness of the predictive model, evaluated through its performance on the 2 validation test sets: AmsterdamUMCdb and MIMIC-IV. The internal validation consists of training and testing on AmsterdamUMCdb, while the external validation involves training the model on matched features between the 2 datasets and then directly testing it on the MIMIC-IV ICU dataset.

**Table 2.** Model performance for cohort A (balanced test set).

Dataset	Metric	**Pre-AF window**		**Non-AF window**	
		AF patients	Non-AF patients	AF patients	Non-AF patients
**AUMC**	Precision	0.75 [0.03]	0.93 [0.02]	NR	NR
	Recall	0.75 [0.07]	0.83 [0.06]	0.83 [0.04]	0.93 [0.02]
	F1	0.75 [0.03]	0.87 [0.00]	NR	NR
	Evaluated measurements	3346	6634	72 653	72 461
	AUC	0.90			
	Accuracy	0.85			
	G-mean	0.79			
**MIMIC**	Precision	0.71 [0.04]	0.91 [0.02]	NR	NR
	Recall	0.73 [0.04]	0.81 [0.04]	0.83 [0.02]	0.91 [0.01]
	F1	0.69 [0.01]	0.87 [0.01]	NR	NR
	Evaluated measurements	6502	9739	184 719	179 387
	AUC	0.86			
	Accuracy	0.83			
	G-mean	0.77			

**Table 3:** Model performance for cohort B (unbalanced test set).

Dataset	Metric	**Pre-AF window**		**Non-AF window**	
		AF patients	Non-AF patients	AF patients	Non-AF patients
**AUMC**	Precision	0.29 [0.05]	0.97 [0.01]	NR	NR
	Recall	0.78 [0.04]	0.79 [0.06]	0.94 [0.01]	0.94 [0.01]
	F1	0.42 [0.04]	0.87 [0.04]	NR	NR
	Evaluated measurements	3226	62 968	82 351	946 961
	AUC	0.92			
	Accuracy	0.88			
	G-mean	0.79			
**MIMIC**	Precision	0.18 [0.03]	0.96 [0.02]	NR	NR
	Recall	0.76 [0.02]	0.79 [0.03]	0.93 [0.01]	0.94 [0.01]
	F1	0.30 [0.01]	0.87 [0.01]	NR	NR
	Evaluated measurements	6502	305 010	184 719	2 111 254
	AUC	0.89			
	Accuracy	0.86			
	G-mean	0.78			

The model reached an AUC of 92% in the unbalanced test set during internal validation (see Table 3, cohort B). With a precision of 29% for AF patients in the Pre-AF period, the model successfully detected 137 patients and identified 78% of AF events. The model demonstrated a high precision of 97% for non-AF patients during the pre-AF period, thereby reducing false alarms. In addition, the model demonstrated consistent performance in the balanced test set (Table 2, cohort A) where precision and recall improved for both AF patients and non-AF patients. Across both cohorts and datasets, the reported G-mean values ranged from 0.77 to 0.79 (Tables 2 and 3), indicating that the model maintains a balanced performance in correctly identifying both AF and non-AF patients, even under class imbalance.

In the external validation, the model’s performance declined slightly in detecting AF, although it remained comparable to the internal validation. On average, AF patients had an LOS of 60.7 hours, and non-AF patients had an LOS of 56.9 hours for the balanced case and 55.8 hours for the unbalanced case. The model achieved an AUC of 0.89 and 0.86, with a G-mean of 0.78 and 0.77 for the unbalanced and balanced test sets, respectively.

### Risk scores over time

**Figure 3.**
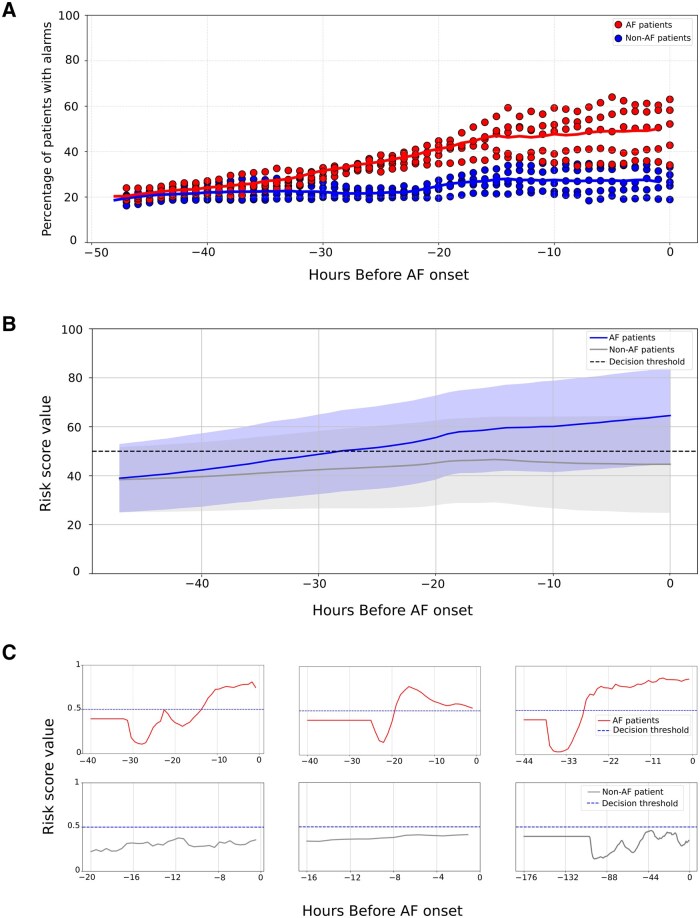
Panel (A) The percentage of patients experiencing at least 1 alarm within the previous 48 hours is displayed, with red dots representing AF patients and blue dots representing non-AF patients. For AF patients, the values correspond to the period preceding the AF event, whereas for non-AF patients they refer to their surrogate AF prediction point. Panel (B) Average risk scores for patients with AF are shown by the blue line, while scores for non-AF patients are represented by the grey line. The shaded areas indicate the standard deviation around the mean for each group. The dashed horizontal line marks the decision threshold of 0.5, used to distinguish between alarm and no-alarm measurements. Panel (C) Individual risk‐score trajectories for a sample of AF and non-AF patients. The red line represents the AF patient, while the gray lines correspond to non-AF patients, with risk scores ranging from 0 to 1 over the last *x* hours prior to the AF event (for the AF patient) or the surrogate AF prediction point (for non-AF patients). The dashed line indicates the decision threshold used to distinguish between alarm and non-alarm classifications.

Figure 3 panel B shows the risk scores predicted by the model for both AF and non-AF patients over the last 48 hours prior to the endpoints. The average risk scores of patients are plotted to illustrate changes over time, with the shaded area indicating the standard deviation around these averages.

For AF patients, the average predicted line (indicated by the solid blue line) shows an upward trend as the time approaches the onset of AF, indicating increased confidence in predicting an AF event. The shaded area around the line indicates the variability in the predictions, with a narrower band suggesting more consistent predictions by the model. In contrast, non-AF patients, represented by the gray line, remain relatively stable and lower throughout the last 48 hours prior to the endpoint, indicating that the model consistently assigns lower risk scores to this group. Individual risk scores for a sample of patients are shown in Figure 3 panel C, with additional examples provided in Figures S1 and S2.

A similar pattern is observed in Figure 3 panel A, where the percentage of AF patients having at least 1 alarm within 48 hours prior to AF onset increases over time and across the folds. In contrast, for non-AF patients, figure shows a different pattern. The percentage of non-AF patients with at least 1 alarm remained below 25% and shows a slight increase in the approximately 20 hours before their surrogate AF prediction point and then stabilizes.

To characterize the lead-time behavior of the model, we identified for each AF patient the earliest time point within the 24-hour Pre-AF window at which the predicted risk crossed the alert threshold and computed the interval between this first alert and AF onset (Figure S3, upper panel), yielding a median lead time of 6.6 hours (±0.05 hour) with alerts spanning nearly the entire 24-hour window. An analogous analysis for non-AF patients, using the end of the observation window as a surrogate endpoint (Figure S3, lower panel), showed a flatter and later alert distribution with a median first-alert time of 15.7 hours (±0.03 hour), indicating lower risk trajectories despite occasional threshold crossings.

### Precision and recall over time

**Figure 4.**
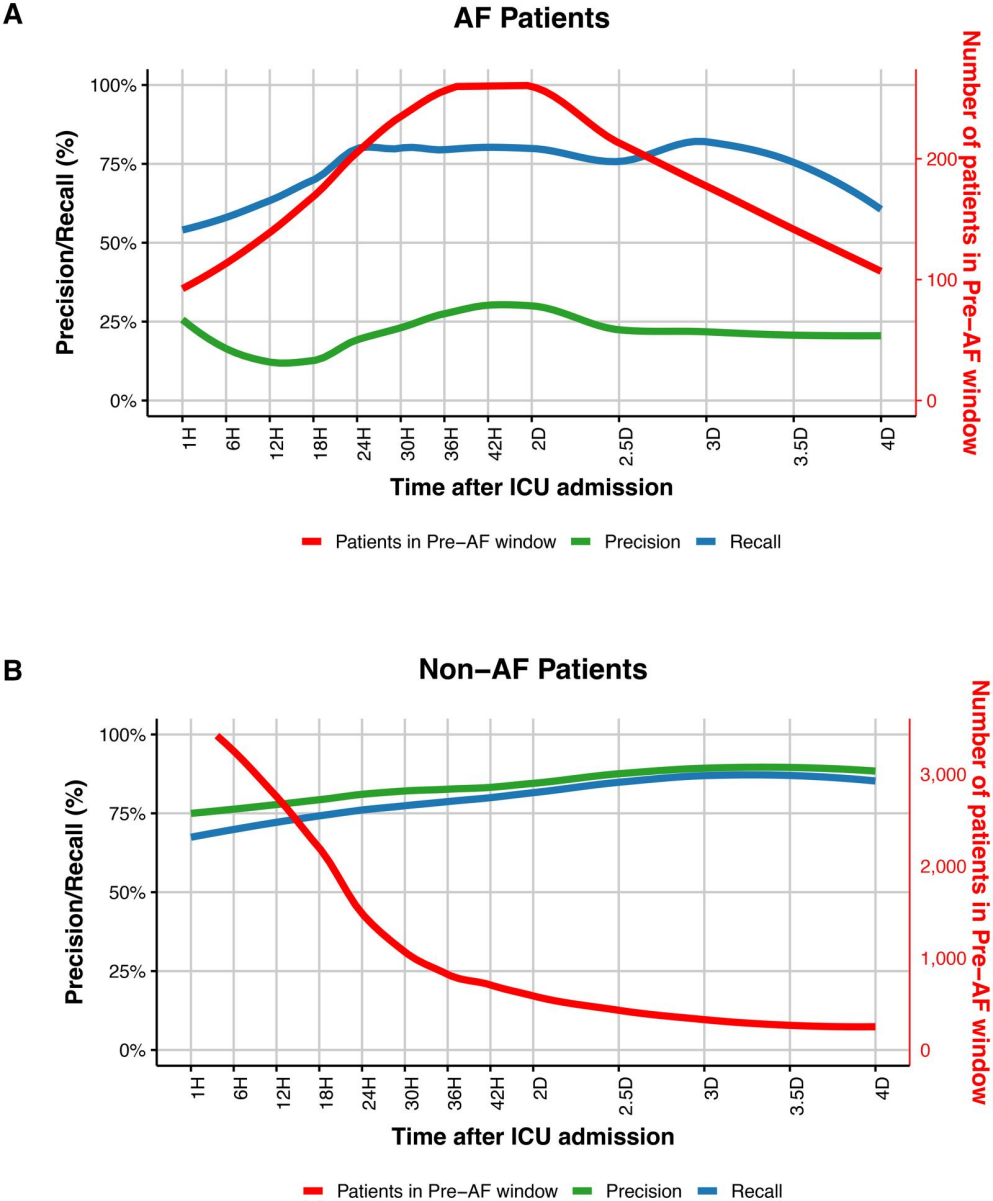
Precision (green) and recall (blue) as a function of time for AF patient at panel (A) and non-AF patients in panel (B). Right *y-*axis showing number of patients in their pre-AF window (red).

Figure 4 shows how recall and precision change over time since admission. The graphs highlight the number of patients in the Pre-AF window and track precision and recall rates from 1 hour to 4 days after ICU admission. For AF patients, more fluctuations were observed. The highest precision performance occurred between 1 and 3 days, notably coinciding with an increase in the number of patients in the Pre-AF window. A similar pattern was observed with recall for this patient group. In contrast, for non-AF patients, both recall and precision remained stable, with a slight increase over time, while the number of patients in the Pre-AF window gradually decreased.

### SHAP analysis

**Figure 5.**
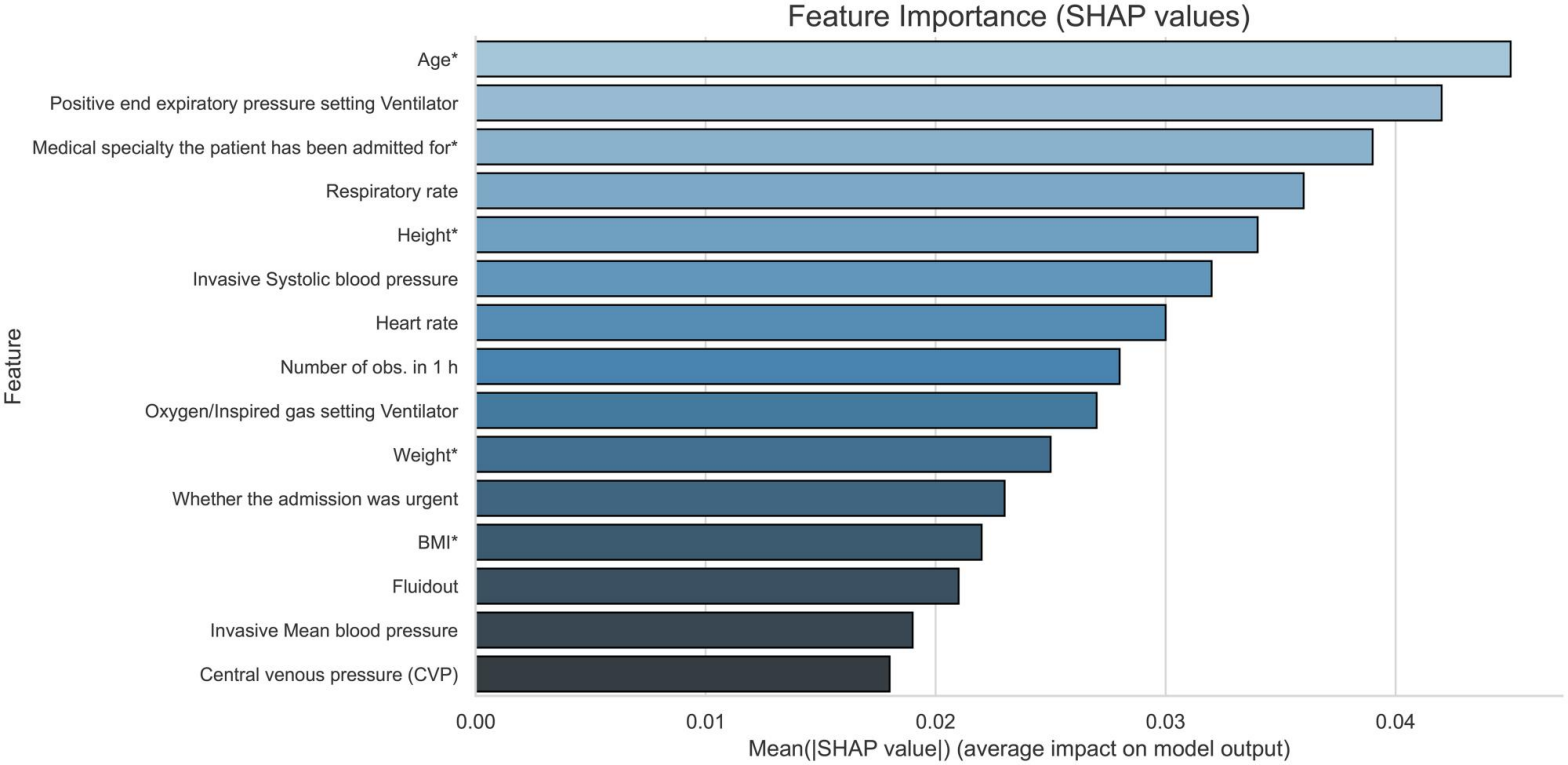
The SHAP values depict the impact of the 15 most important features on the model’s risk score prediction. The bars represent the significance of each feature in predicting AF. SHAP values for each feature were calculated by taking the absolute values and then averaging them to determine their contribution. Features marked with an asterisk (*) denote static variables..

The SHAP summary plot provides insight into the feature importance determined by the predictive model (Figure 5). Key clinical features such as age, Positive End-Expiratory Pressure, Medical specialty (medical specialty for which the patient has been admitted), and Respiratory rate emerged as strong influencers on the model’s risk score prediction. The spread of mean absolute SHAP values for each feature indicates the variability of their impact on the predictions, with age being notably significant.

**Figure 6.**
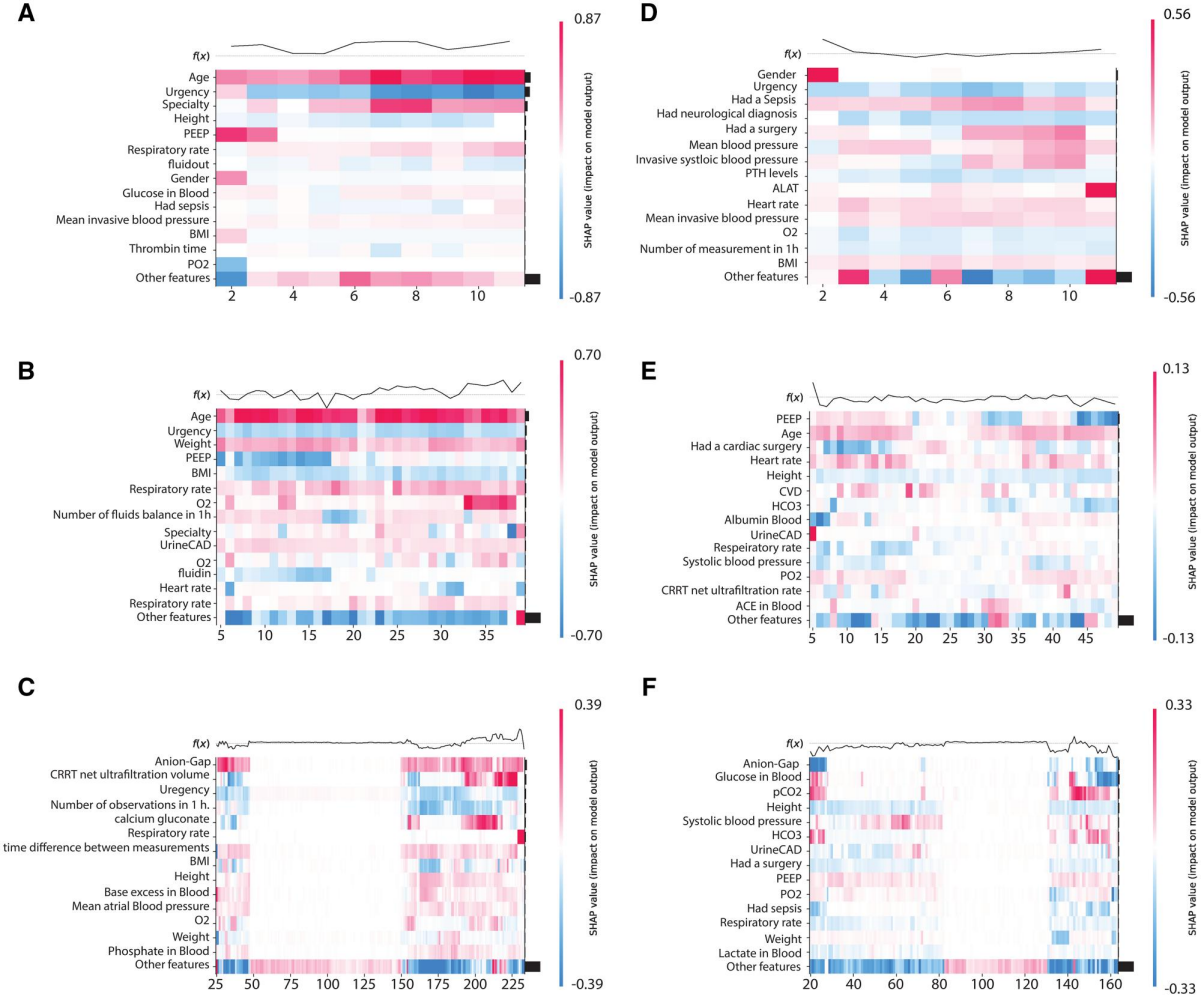
SHAP values for different AF patients are shown for those with LOS < 24, LOS between 24 and 100, and LOS > 100 in panels (A), (B), and (C), respectively, while panels (D), (E), and (F) represent non-AF patients for the same LOS categories. f(x) is the aggregated SHAP value for time *x*.

At the patient level, we also examined SHAP values for AF and non-AF patients categorized into short, medium, and long LOS, depicted in Figure 6. In each panel, the SHAP heatmap illustrates how different factors impact the model’s predictions for each patient. Red indicates features that raise the predicted risk, while blue shows those that lower it. The figure also demonstrates that the most important factors vary from patient to patient, with the influence of each feature differing in magnitude.

## Discussion

We conducted a retrospective study to evaluate the performance of a real-time prediction model designed to predict the onset of AF early in ICU patients. The model was tested internally on the AmsterdamUMCdb and externally on the MIMIC-IV dataset, using both balanced and unbalanced test sets. It achieved an AUC score of 92%, with a precision value of 29% for AF patients and 89% for non-AF patients in the unbalanced test set. Compared to similar studies using the same dataset, our results demonstrated improvements across the evaluated metrics. Verhaeghe et al. implemented a semi-real-time model and reported an AUC of 0.81, AF patient precision of 0.07, and recall of 0.77. Ortega-Martorell et al., using a patient-wise logistic regression model, also reported an AUC of 0.81.^5^^,^^8^

Compared with Ortega-Martorell et al., our deep learning model was better able to capture complex, non-linear patterns in the data, which traditional methods might miss due to their limited capacity for modeling such relationships. Additionally, unlike both Ortega-Martorell et al. and Verhaeghe et al., our dataset spans from patient admission through to the point at which AF develops. This extended observation period enables us to capture trends in both Non-AF and Pre-AF windows, which were not fully represented in their studies. This richer temporal information enhanced the model’s ability to learn from the progression of AF and improved its predictive performance. In addition, the use of a meta-learning framework may have contributed to the improved performance metrics by potentially enhancing the generalization of model parameters across patients within the sample. However, it is also possible that the observed improvements are partially or primarily due to the longer observation time included in our dataset.

External validation showed a similar trend, although with lower performance metrics. In addition, the predicted risk scores for AF patients were generally higher compared to those for non-AF patients. An analysis of the most critical features, based on SHAP values, identified age, positive end-expiratory pressure, medical specialty (the specialty for which the patient was admitted), and respiratory rate as key determinants. In a real-world ICU, the model would run continuously from admission onward, updating the hourly AF risk score as new numerical measurements become available. Because the model is trained within a meta-learning framework, patient-level adaptation occurs implicitly as it conditions on each individual’s evolving time series, without requiring manual recalibration at the point of care. Clinically, these risk scores and the resulting alerts could be integrated into existing electronic dashboards, with thresholds tailored to each ICU to balance early detection against alarm burden.

The study has several limitations. First, it was not possible to identify patients who developed AF after discharge. Additionally, the diagnosis of AF relies on nursing charts, as electrocardiograms were not available in the AmsterdamUMCdb.^5^ In routine clinical care, these chart entries are typically based on ECG monitoring, so ECG remains the reference standard used to define the outcome, whereas our prediction model uses only numerical data as predictors. This assumes that nurses’ recordings are accurate and timely to avoid data leakage, generally capturing clinically significant AF episodes, but not all. Machine-learning models using ECG waveforms can improve AF prediction and diagnosis speed, and incorporating ECG data may enhance the current model. However, ECG-based prediction models require annotation of extended pre-event ECG segments, which limits feasibility at scale. Our results therefore complement ECG-based approaches by demonstrating that meaningful early risk stratification is achievable even when only routinely collected numerical data are available. Future studies should explore whether ECG integration improves the model, though this may increase false positives due to noise. ECG signals are sensitive to movement and artifacts, limiting their practicality for AF prediction. Second, in the unbalanced test set, while the model achieves higher AUC and precision than earlier studies, the precision may still lead to alarm fatigue due to the low incidence rate of AF. Our current approach addresses imbalance during training through the Combo Loss; however, additional gains may be achieved by adjusting the decision threshold at inference time.^13^ Quantile-based thresholding strategies, which adapt the threshold to the expected prevalence of the minority class, could further reduce false alarms and improve clinical usability.^14^

An additional direction for future research is to incorporate structured admission-time clinical context to provide an explicit baseline AF risk prior to longitudinal monitoring. For example, demographics, comorbidities, and prior rhythm history (when available) could be used directly, or summarized via AF-risk prediction scores such as CHARGE-AF or C_2_HEST. While such priors may improve early calibration and reduce the amount of monitoring required before reliable predictions can be made, these scores were largely developed outside ICU settings and would therefore require validation and recalibration.^15^

During harmonization, we averaged each feature’s measurement frequency per patient to a uniform hourly rate, also recording the original count of measurements. However, this approach might result in the loss of valuable information. The varying number of measurements reflects clinical decisions in the ICU over which we had no control. The impact of these varying frequencies on the model’s output remains unclear, potentially introducing bias due to the intensity of monitoring, referred to as bias-by-indication. Although the model updates predictions hourly, this choice reflects a practical compromise rather than a fixed physiological assumption. In these real-world ICU databases, measurement frequencies vary substantially across features, and sub-hourly windows would introduce extensive missingness requiring heavy imputation, whereas longer windows would suppress clinically relevant temporal fluctuations. Higher-resolution datasets with consistent sampling across all variables will be needed to meaningfully explore sub-hourly prediction horizons in future work, particularly for the most influential dynamic features such as heart rate, respiratory rate, invasive blood pressures, ventilator settings, and fluid balance. Specifically, changes in patients’ predicted risk score patterns, often preceding AF, are key model features. Consequently, the model might underperform when healthcare providers have not recognized AF signs, undermining its effectiveness as a preemptive alert system. This issue is particularly relevant for medications prescribed in response to specific patient concerns.

We assumed that early signs of AF occur within 24 hours before onset. This window was necessary for training and evaluating the algorithm. However, shorter windows create a more challenging task due to increased dataset imbalance and reduced data availability for learning AF progression. A minimum LOS requirement of 6 hours was set to ensure sufficient measurements for labeling patients as AF or non-AF. This limits applicability to patients developing AF early during their ICU stay. Alternative methods should be explored to include these patients in training and testing. One approach is to generate synthetic data for patients with shorter LOS to augment the dataset and expose the algorithm to a broader range of LOS.

## Conclusion

Our research focused on developing a real-time AF prediction model using a meta-learning approach. We trained and evaluated the model on the AmsterdamUMCdb dataset, achieving a high AUC of 92% and a precision of 29%, demonstrating its effectiveness in distinguishing between patients at risk for AF and those not at risk. While these results demonstrate potential for future clinical benefits, further refinement in its ability to identify patients at risk for AF is necessary before clinical implementation.

## Supplementary Material

ooag110_Supplementary_Data

## Data Availability

The code is publicly available on GitHub at: https://github.com/Mehranmzn/AF_MAML. The AmsterdamUMCdb dataset is available upon request; see: https://github.com/AmsterdamUMC/AmsterdamUMCdb and https://amsterdammedicaldatascience.nl. The MIMIC-IV dataset is available upon request via PhysioNet: https://physionet.org/content/mimiciv/2.1/.
