# Supplementary material for: Real-time prediction of atrial fibrillation in intensive care unit: a meta-learning approach
Source: JAMIA Open. 2026 Jul 11;9(4):ooag110. doi: 10.1093/jamiaopen/ooag110 (PMC13355586; doi:10.1093/jamiaopen/ooag110)
Supplement: ooag110_Supplementary_Data [file ooag110_supplementary_data.zip › Submission_of_Atrial_fibrillation_prediction_using_Meta_learning-SUPPLEMENTAL.pdf]

A Supplemental to "Real-time prediction of atrial fibrillation in intensive care unit (ICU): A meta-learning approach"

A.1 Variable merging

A.2 Missing data imputation

We have implemented two procedure to impute missing data. If a column has more than 90% of its values as missing, we replace those values with global mean, and if it is less, we use multiple imputation by chain equation (MICE). The columns that we used MICE for imputation are:

- Invasive mean arterial pressure
- Invasive mean systolic pressure
- Respiratory rate
- Respiratory rate monitor
- Heart rate
- Inspired oxygen concentration measurement
- Inspired oxygen concentration setting
- Positive-end expiratory pressure setting

A.3 Removing artifacts

In the following variables, we used the provided dictionary from AmsterdamUMCdb to eliminate variables that contain artifacts or implausible values:

Table 1: Minimum and maximum expected values of five features to remove artifacts and implausible values. Values obtained from original documentation of AmsterdamUMCdb

**Measurement**	**Minimum Expected Value**	**Maximum Expected Value**
Invasive Mean blood pressure (mmHg)	30	250
Invasive Mean blood pressure II (mmHg)	30	250
Invasive Systolic blood pressure (mmHg)	40	250
Cardiac Output (L/Min)	0	17
Heart rate (bpm)	1	250

A.4 List of features

List of features alongside their itemid in the AmsterdamUMCdb is provided in Tables tables 2 to 5. To get the required features for the analysis we followed same feature selection as Verhaege et.al.

Table 2: Summary of Features: Vitals, Monitoring and Outputs

Measurement (Unit)	ItemID	Missingness	Imputation method
ABP gemiddeld	6642	11.0%	MICE
ABP systolisch	6641	11.0%	MICE
Ademfreq.	13092	52.5%	Forward fill per patient
Bloed-flow	10736	96.3%	Forward fill per patient
Buikomvang	12087	97.1%	Forward fill per patient
Cardiac Output	6656	81.2%	Forward fill per patient
Hartfrequentie	13075	1.9%	MICE
PEEP (Set)	12284	52.4%	Forward fill per patient
PiCCO CO (Cardiac Output)	13151	95.9%	Forward fill per patient
Apache II Hoofdgroep	18588	93.7%	Forward fill per patient
UrineIncontinentie	8800	91.7%	Forward fill per patient
Zuurstof toediening	18587	93.1%	Forward fill per patient
Temp Huid	13063	90.6%	Forward fill per patient
O2 l/min	8845	93.8%	Forward fill per patient
O2-Saturatie (bloed)	12311	92.7%	Forward fill per patient
UrineSpontaan	8798	92.4%	Forward fill per patient
Bloeddruk gemiddeld	12123	94.5%	Forward fill per patient
O2-Saturatie (urine)	10191	93.3%	Forward fill per patient
Zuurstof saturatie (bloed)	10208	92.9%	Forward fill per patient

Abbreviations: ABP = Arterial Blood Pressure; PEEP = Positive End-Expiratory Pressure; PiCCO = Pulse Contour Cardiac Output; O2 = Oxygen; MICE = Multiple Imputation by Chained Equations.

Table 3: Summary of Features: Medications and Injections

Measurement (Unit)	ItemID	Missingness	Imputation method
Actrapid (Insuline)	7624	94.2%	0 (no administration)
Amiodaron Oplaaddosis	6844	97.8%	0 (no administration)
Amiodaron Onderhoudsdosis	9015	98.0%	0 (no administration)
Dobutamine (Dobutrex)	7178	96.1%	0 (no administration)
Dopamine (Inotropin)	7179	96.8%	0 (no administration)
Fentanyl	7219	92.4%	0 (no administration)
Furosemide (Lasix)	7244	95.4%	0 (no administration)
Midazolam (Dormicum)	7194	92.9%	0 (no administration)
Morfine	7225	93.3%	0 (no administration)
Noradrenaline (Norepinefrine)	7229	96.5%	0 (no administration)
Velosuline (Insuline)	9014	94.7%	0 (no administration)
Sotalol (Sotacor)	7006	98.3%	0 (no administration)
Atenolol (Tenormin)	6862	98.2%	0 (no administration)
Amiodaron	16113	97.6%	0 (no administration)
Bisoprolol	19368	97.9%	0 (no administration)
Atropine sulfaat	6864	97.0%	0 (no administration)
Adenosine	6816	97.2%	0 (no administration)
Natrium bicarbonaat 8,4%	7295	96.7%	Forward fill per patient
Verapamil (Isoptin)	7139	98.5%	0 (no administration)
Carvedilol (Eucardic)	12938	98.4%	0 (no administration)
Calciumgluconaat	19164	97.1%	0 (no administration)
Flecainide (Tambocor)	7224	98.7%	0 (no administration)
Calcium Glubionaat	7412	96.9%	Forward fill per patient
Metoprolol (Selokeen)	7184	98.1%	0 (no administration)
Terlipressine (Glypressin)	12467	97.3%	0 (no administration)
Bumetanide (Burinex)	6882	95.9%	0 (no administration)
Procainamide (Pronestyl)	6927	98.9%	0 (no administration)
Nebivolol	19138	98.6%	0 (no administration)
Phenytoin (bloed)	9973	98.1%	Forward fill per patient
Dopamine (bloed)	15640	95.9%	Forward fill per patient
Adrenaline (Epinefrine)	10197	96.9%	0 (no administration)

Table 4: Summary of Features: Laboratory Measurements (Part A). All missing laboratory values were imputed using forward fill per patient.

**Measurement (Unit)**	**ItemID**	**Missingness**
ALAT	6800	96.2%
ACTH (bloed)	10195	98.4%
APTT (bloed)	11944	95.7%
ASAT	6806	96.3%
Act.HCO3 (bloed)	9992	92.7%
Alb.Chem (bloed)	9937	94.5%
Alk. Fosfatase	6803	96.5%
Alk.Fosf. (bloed)	11984	96.4%
Anion-Gap (bloed)	9559	93.0%
Bezinking (bloed)	11902	95.4%
Bili Totaal	6813	96.1%
Bilirubine geconjugeerd	6812	96.0%
Ca++(7.4) Astrup	9561	93.2%
Calcium	6817	94.3%
Calcium totaal (bloed)	9933	94.4%
Chloor	6819	94.6%
Chloor (bloed)	9930	94.7%
Cholesterol (bloed)	9954	95.1%
Cortisol (bloed)	10238	98.2%
CRP (bloed)	10079	95.2%
D-dimeren (bloed)	10393	96.1%
Ery's (bloed)	9962	95.6%
Fibrinogeen (bloed)	10175	95.8%
Glucose (bloed)	6814	93.8%
Kalium (bloed)	13070	94.1%
Kreatinine (bloed)	6818	94.2%
Leuco's (bloed)	9963	95.5%
Natrium (bloed)	9924	94.0%
PCO2 (bloed)	9990	93.1%
ph (bloed)	12310	92.9%
Prothrombinetijd (bloed)	11893	95.9%
Serum Totaal Eiwit (bloed)	10051	94.8%
Thrombo's (bloed)	9964	95.3%
TroponineT (bloed)	10407	96.4%
Ureum (bloed)	9943	94.6%
Vrij-T3 (bloed)	10201	98.0%
Vrij-T4 (bloed)	10187	98.1%
Y-GT (bloed)	12071	96.6%
Albumine (bloed)	11903	94.9%
AnGap	8492	93.3%
Ca-ion	9560	92.8%
B.E (bloed)	9994	92.6%
CK (bloed)	11998	95.9%
CK-MB	6824	96.0%
Thrombinetijd (bloed)	11950	95.6%
TSH (bloed)	11925	98.3%

Abbreviations: ALAT = Alanine aminotransferase; ASAT = Aspartate aminotransferase; APTT = Activated partial thromboplastin time; ACTH = Adrenocorticotropic hormone; CRP = C-reactive protein; CK = Creatine kinase; CK-MB = CK–MB fraction; TSH = Thyroid-stimulating hormone; Vrij-T3 = Free T3; Vrij-T4 = Free T4; Y-GT = Gamma-glutamyl transferase; B.E. = Base excess; AnGap = Anion gap; Ca-ion = Ionized calcium.

Table 5: Summary of Features: Laboratory Measurements (Part B). All missing laboratory values were imputed using forward fill per patient.

Measurement (Unit)	ItemID	Missingness
Thrombinetijd (bloed)	11951	95.7%
Serum Eiwit (bloed)	8908	94.7%
HCO3 (bloed)	11949	92.9%
Gamma GT	12072	96.4%
Protrombinetijd	6789	95.8%
Vrij Cortisol (verz. urine)	11941	98.3%
AChE (bloed)	7001	97.4%
ACTH (plasma)	10092	98.6%
Ammonia (bloed)	10144	96.1%
Amylase (bloed)	10146	95.6%
Antitrombine III (bloed)	10153	96.2%
Aspartaat aminotransferase (AST)	10155	96.2%
Base Excess (bloed)	10157	92.7%
Bicarbonate (HCO3) (bloed)	10159	93.0%
Bloedplaatjes (bloed)	10161	95.2%
BNP (bloed)	10162	96.3%
Calcium geïoniseerd	10357	94.3%
Cortisol vrij (urine)	10164	98.4%
Creatine kinase	10165	95.9%
Creatinine clearance (urine)	10166	94.8%
Ferritine (bloed)	10169	96.0%
Fosfaat anorganisch	10171	94.6%
Glucose (urine)	10173	94.2%
Hb (bloed)	10174	95.1%
HDL-cholesterol	10176	95.0%
Hemoglobine (bloed)	10177	95.3%
IGF-1 (bloed)	10179	98.8%
Insuline (bloed)	10180	98.1%
Lactaat (bloed)	10181	93.8%
Lipase (bloed)	10183	95.5%
LDL-cholesterol	10184	95.2%
Magnesium (bloed)	10186	94.9%
Noradrenaline (bloed)	10188	97.0%
Osmolaliteit (bloed)	10189	94.5%
Parathormoon (PTH) (bloed)	10192	98.2%
pCO2 (urine)	10194	93.2%
Prostaat Specifiek Antigeen (PSA)	10196	98.7%
Serum Glucose	10197	94.0%
T4 Vrij	10198	98.0%
TSH (urine)	10199	98.4%
T4 Totaal	10200	98.1%
Troponine-I (bloed)	10202	96.5%
Ureum (urine)	10203	94.4%
Vitamine B12 (bloed)	10205	97.6%
Vitamine D (25-OH)	10206	97.9%
Zink (bloed)	10209	97.1%
Oestradiol (bloed)	10210	98.5%
pH (urine)	10212	93.1%
Bilirubine direct	10213	96.2%
Serum ijzer (bloed)	10215	94.7%
Ketonen (urine)	10217	94.3%
Glycated Hemoglobine (HbA1c)	10218	96.8%
Albumine/Creatinine-ratio (urine)	10219	95.2%
Troponine	8115	96.2%

Abbreviations: AChE = Acetylcholinesterase; ACTH = Adrenocorticotropic hormone; AST = Aspartate aminotransferase; BNP = B-type natriuretic peptide; Hb = Hemoglobin; HbA1c = Glycated hemoglobin; HDL = High-density lipoprotein; LDL = Low-density lipoprotein; IGF-1 = Insulin-like growth factor 1; PTH = Parathyroid hormone; PSA = Prostate-specific antigen; Vit D (25-OH) = 25-hydroxyvitamin D.

A.5 matched features

For external validation, we performed feature matching because not all features were present in both datasets, and some features could not be directly translated between them. Hence, a subset of features at are matched used for retraining the AmsterdamUMCdb and then tested on MIMIC-IV. Matched features that used for training and testing are provided in table 6.

Table 6: Matched features of AmsterdamUMCdb and MIMIC-IV

abp gemiddeld	magnesium
abp systolisch	hemoglobine
laktaat	cvd
o2-saturatie (bloed)	fosfaat
pco2 (bloed)	a_fio2
ph (bloed)	o2 l/min
po2	peep (set)
ademfreq.	noradrenaline (norepinefrine)
time_diff	propofol (diprivan)
fluidin_h	fluidout
hartfrequentie	calciumgluconaat 10%
height	count
kaliumchloride (kcl)	sepsis_bool
cardiac_surg_bool	cardio_surgery_new
age	gender_category
weight	BMI
N.measurement in an hour	

A.6 Loss function and MAML:

The `ComboLoss` combines the Binary Cross-Entropy (BCE) loss and Dice loss, useful for handling both class imbalance and overlap of predicted and actual values. This is commonly applied in binary classification and segmentation tasks.

Binary Cross-Entropy (BCE) Loss:

The BCE loss is computed as:

$$\text{BCE\_Loss}(p, y) = -\frac{1}{N}\sum_{i=1}^{N}\left[y_i \log(p_i) + (1 - y_i)\log(1 - p_i)\right]$$

where p is the predicted probability and y is the target binary label. The ‘pos_weight‘ parameter modifies the contribution of the positive class in the loss function to address class imbalance.

Dice Loss:

The Dice coefficient is used to measure the overlap between two samples, calculated as:

$$\text{Dice\_Loss} = 1 - \frac{2 \cdot \text{intersection} + \text{smooth}}{\text{sum of predictions} + \text{sum of targets} + \text{smooth}}$$

where the intersection is the element-wise multiplication of the predicted and target values.

Combo Loss:

The final loss function is a combination of the BCE loss and the Dice loss:

$$\text{Combo\_Loss} = \text{BCE\_Loss} + \beta \cdot \text{Dice\_Loss}$$

where β is a scaling factor that controls the relative importance of the Dice loss in the final combined loss. $\beta$is a hyper-parameter that is optimized during training.

The loss for task T_i is obtained as :

$$\mathcal{L}_{T_i}(f_\theta) = \frac{1}{|Q_i|} \sum_{(x,y)\in Q_i} \text{ComboLoss}(f_{\theta'}(x), y) \quad (1)$$

where θ represents the initial parameters of the model and θ' represents the updated parameters after training on the support set S_i.

The update rule for the inner loop is given by:

$$\theta' = \theta - \alpha \nabla_\theta \mathcal{L}_{S_i}(f_\theta) \quad (2)$$

Here, α is the inner loop learning rate, and $\mathcal{L}_{S_i}(f_\theta)$ is the loss on the support set using ComboLoss.

Building upon the fitted model from the inner loop updates, the MAML framework is designed to enable rapid model adjustments to new patient data, instead of specializing in a single, specific task. This is achieved by monitoring the loss function values on the query set, referred to as *outer loop updates.* The primary goal of MAML in this context is to refine the base learner LSTM's initial parameters to ensure that these parameters are sufficiently generalized across a variety of patient profiles. The MAML objective function can be represented as:

$$\min_\theta \sum_i \mathcal{L}_{T_i}(f_{\theta'}) \quad (3)$$

and the MAML update rule is:

$$\theta \leftarrow \theta - \beta \nabla_\theta \sum_i \mathcal{L}_{T_i}(f_{\theta'}) \quad (4)$$

Where β is the outer loop learning rate. The complete loss function for the MAML in our study, incorporating the inner and outer updates, can be expressed as:

$$\mathcal{L}_{\text{meta}}(\theta) = \sum_i \mathcal{L}_{Q_i}(f_{\theta - \alpha \nabla_\theta \mathcal{L}_{S_i}(f_\theta)}) \quad (5)$$

A.7 Individual risk scores

Figures 1–2 represents individual risk scores generated by the model for sample of AF and non-patients.

A.8 Training and Hyper-parameter optimization

For the training, the Adam optimizer is selected for its ability to adjust learning rates adaptively, in conjunction with a neural network Binary Cross-Entropy with Log Loss function that applies varying weights to different classes to address class imbalances. To enhance the training of our model, we undertook hyper-parameter optimization. Details provided in table 5a.

**Parameter**	**Minimum Value**	**Maximum Value**	**Selected Parameter**
Number of hidden layers	1	5	2
Number of epochs	10	200	50
β coefficient in combo loss	1	10	5
Number of LSTM units	16	512	32
Number of inner loop updates for meta-learning	1	10	2
Learning rate for inner loop	0.0001	0.001	0.0001
Learning rate for outer loop	0.0001	0.001	0.001
Number of tasks in meta-learning	5	20	10
Number of AF patients in each task	100	1000	300
Number of non-AF patients in each task	100	1000	300
Dropout rate	0.1	0.5	0.3

Table 6: MAML-LSTM parameters checked for hyperparameter optimization

Several models were tested to select the best outcome. The models include traditional architectures such as LSTM and GRU, as well as more advanced configurations like LSTM and GRU models combined with attention mechanisms. Additionally, TCN (Temporal Convolutional Networks) and CNN (Convolutional Neural Networks) were also evaluated. The performance of each model was measured using the Area Under the Curve (AUC) metric on the internal unbalanced test set, as shown in the table below:

**Model Name**	**AUC**
LSTM	0.92
GRU	0.89
LSTM + Attention Layers	0.72
TCN	0.75
CNN	0.74
GRU + Attention Layers	0.73

Table 8: Comparison of AUC scores for different models

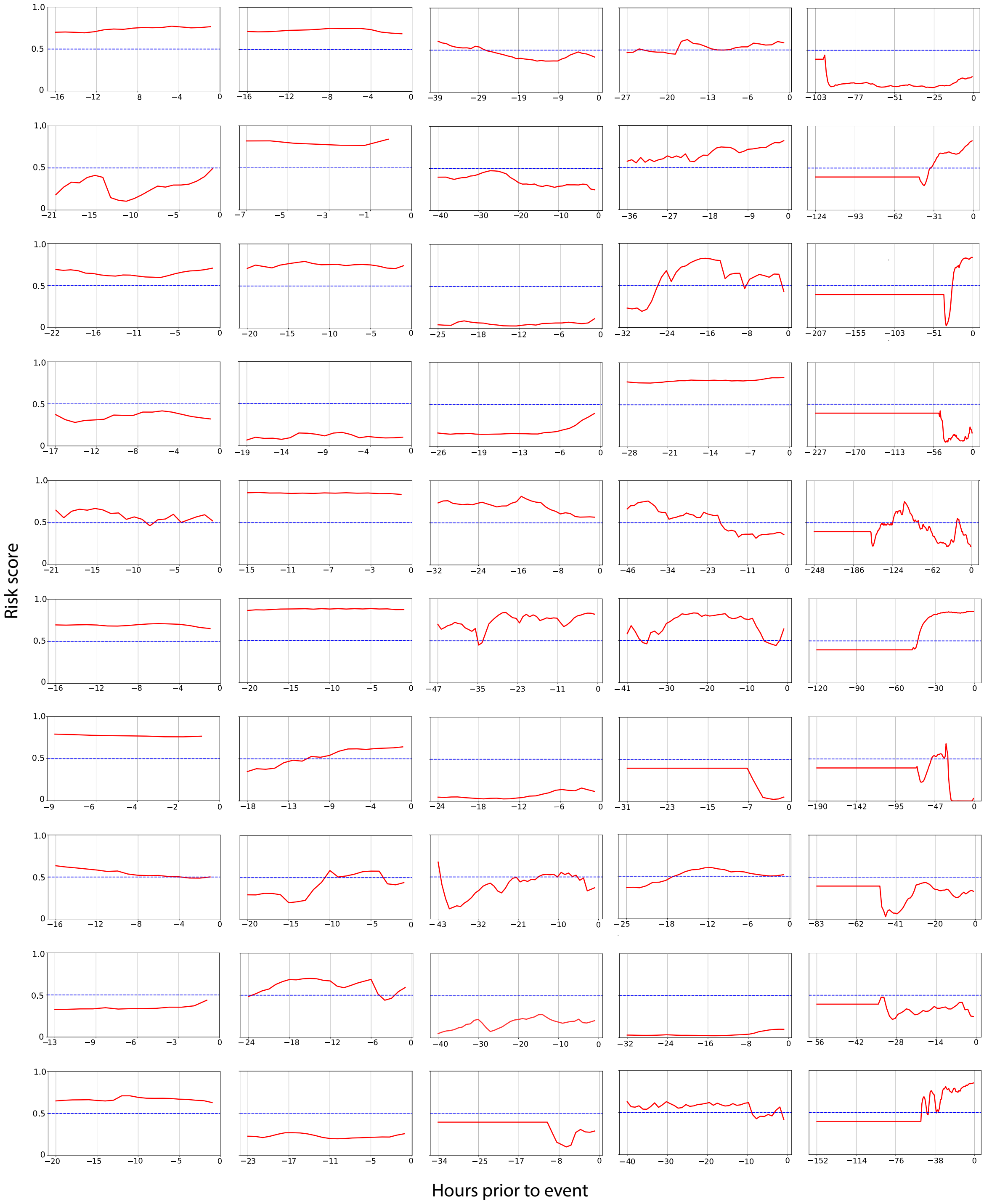

Figure 1: Individual risk scores for a sample of AF patients are shown, with the red line representing the risk score values (between 0 to 1) over the last x hours prior to the onset of AF. The dashed line indicates the decision threshold used to distinguish between alarms and measurements without alarm

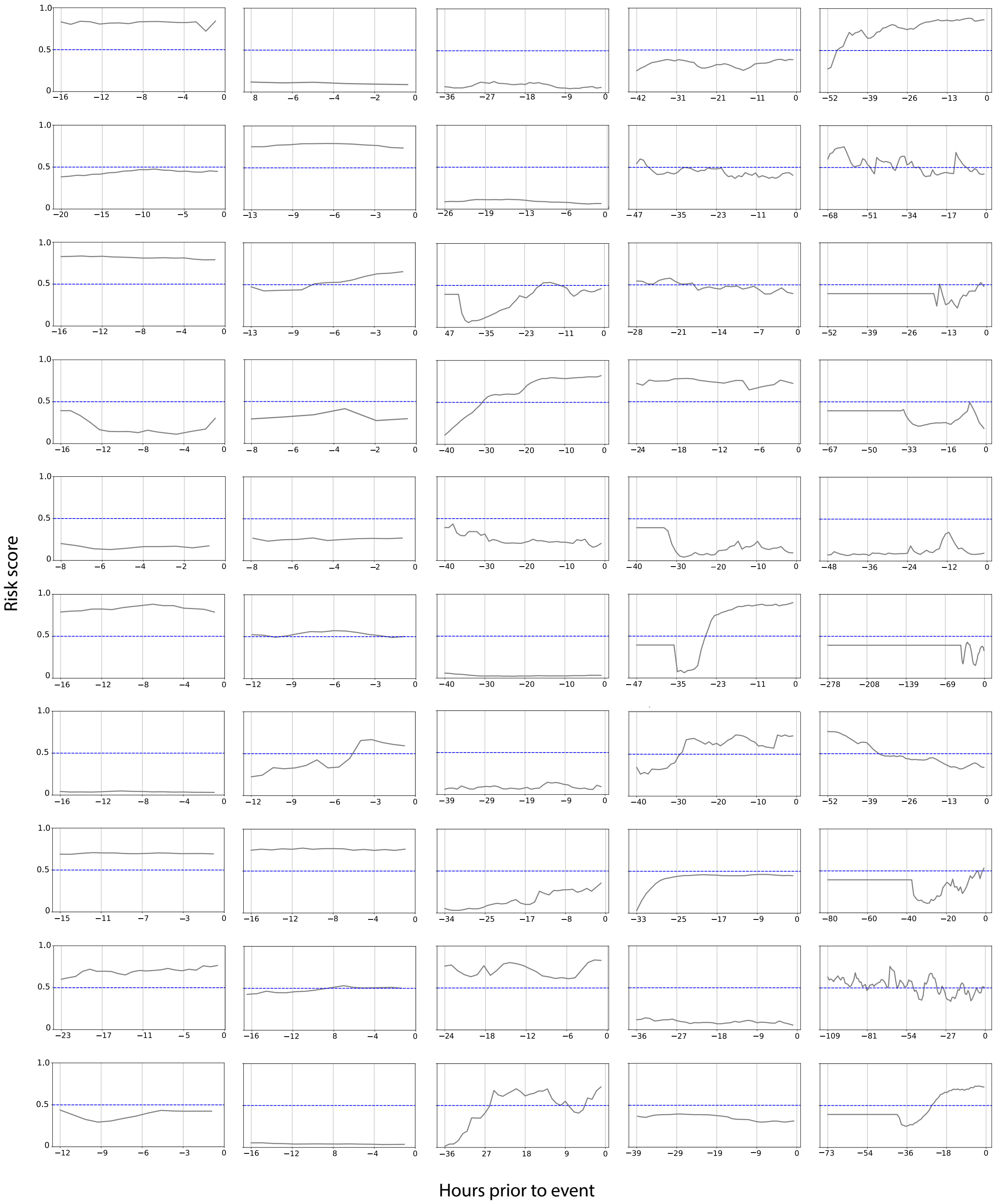

Figure 2: Individual risk scores for a sample of non-AF patients are shown, with the grey line representing the risk score values (between 0 to 1) over the last x hours prior to the surrogate AF time point. The dashed line indicates the decision threshold used to distinguish between alarms and measurements without alarm

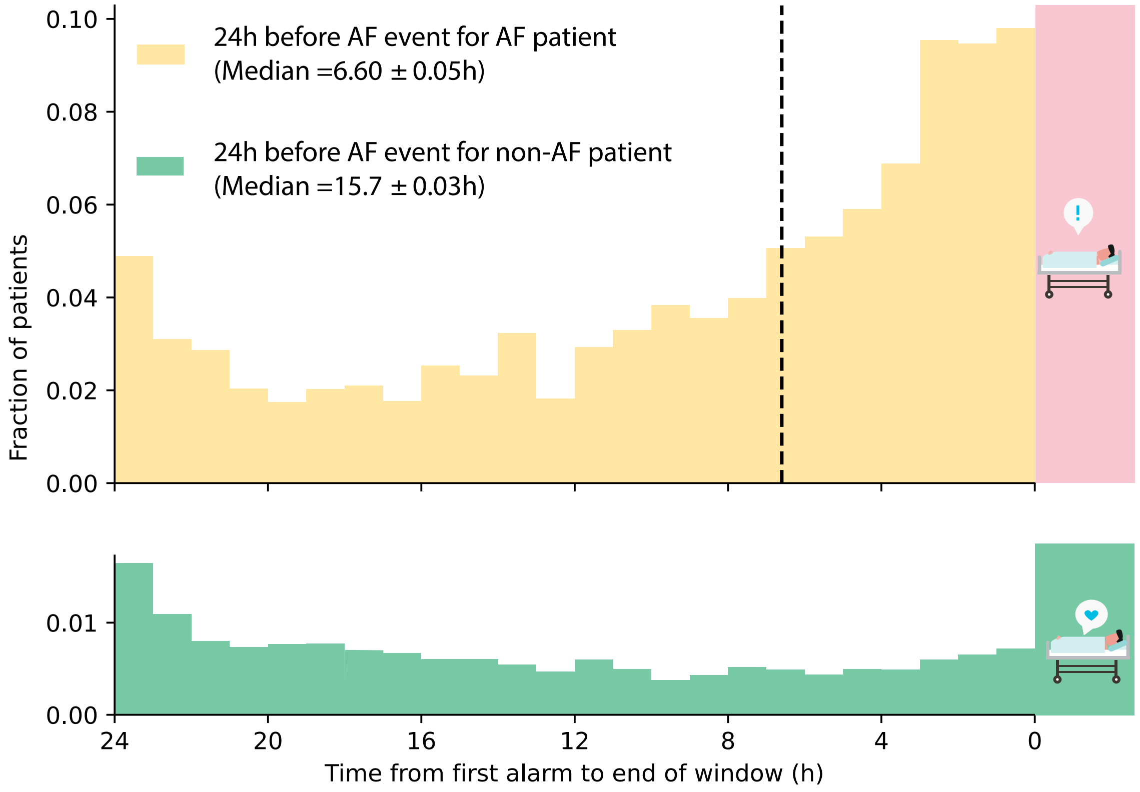

Figure 3: Each bar represents the fraction of patients whose first alarm occurred within a given 1-hour interval during the 24 hours preceding the endpoint. AF patients are shown in yellow in the upper panel, and non-AF patients are shown in green in the lower panel. The dashed vertical line indicates the median time of the first alarm for AF patients. For non-AF patients, the end of the 24-hour observation window is used as a surrogate endpoint. Twenty-two percent of AF patients did not have at least one alarm during the 24-hour window, whereas 21% of non-AF patients had at least one alarm within the same window.
